# Epigenetic analyses of the insulin-like growth factor binding protein 1 gene in type 1 diabetes and diabetic nephropathy

**DOI:** 10.1186/1868-7083-6-10

**Published:** 2014-05-30

**Authors:** Tianwei Gu, Henrik Falhammar, Harvest F Gu, Kerstin Brismar

**Affiliations:** 1 Rolf Luft Research Center for Diabetes and Endocrinology, Department of Molecular Medicine and Surgery, Karolinska Institutet, Stockholm, Sweden; 2 Department of Endocrinology, Metabolism and Diabetes, Karolinska University Hospital, Stockholm, Sweden

**Keywords:** IGFBP1, DNA methylation, Type 1 diabetes, Diabetic nephropathy

## Abstract

**Background:**

Clinical observations have demonstrated that high levels of circulating insulin-like growth factor binding protein-1 (IGFBP-1) are associated with type 1 diabetes (T1D), whereas low serum IGFBP-1 levels are associated with the risk of type 2 diabetes (T2D). Recently, we reported that increased DNA methylation levels in the *IGFBP1* gene were associated with T2D. In the present study, we evaluated the epigenetic changes of *IGFBP1* in T1D and diabetic nephropathy (DN).

**Results:**

In total, 778 Swedish individuals, including T1D patients with or without DN and subjects with the normal glucose tolerance (NGT), were involved in the study. *IGFBP1* methylation levels in genomic DNA extracted from peripheral blood were analyzed with bisulfite pyrosequencing. Serum IGFBP-1 levels were measured with radioimmunoassay. We found that DNA methylation levels in the *IGFBP1* gene were decreased (15.6% versus 16.9%; *P* < 0.001), whereas serum IGFBP-1 levels were increased (31 versus 24 μg/L, *P* = 0.003) in T1D patients compared with NGT subjects. Furthermore, T1D patients with DN had increased circulating IGFBP-1 concentration compared with the patients without DN (52 versus 28 μg/L; *P* = 0.006). However, no difference of the *IGFBP1* DNA methylation levels between T1D patients with and without DN was observed.

**Conclusions:**

This study shows for the first time that T1D patients had decreased DNA methylation levels in the *IGFBP1* gene and further implies that increased circulating IGFBP-1 levels are associated with T1D and DN.

## Background

Type 1 diabetes (T1D) is an autoimmune disorder characterized by the absence of insulin production, and the patients with T1D require insulin therapy to sustain life. T1D is the most common type of diabetes among children and young adults, but its onset can occur at any age [1]. T1D patients usually develop complications gradually over the years. Diabetic nephropathy (DN) is one of the most common microvascular complications of diabetes, affecting approximately 40% of individuals with type 1 diabetes [2]. The patients with DN usually start with microalbuminuria and then develop persistent proteinuria, hypertension and declined kidney function. T1D and related DN are complex diseases that result from the combination of genetic and nongenetic factors. In recent years, epigenetic modifications were considered to be involved in the pathogenesis of diabetes and diabetic complications [3-5]. DNA methylation changes are involved in epigenetic regulation mechanisms, which allow alteration of gene function without mutating the sequence. DNA methylation analyses can be performed in the scales of global genome or specific gene regions and in the material of peripheral blood with mixed cell types, particular cell types, or in target tissues [6-8]. Dick *et al.*[9] recently used both approaches of whole-blood DNA methylation profiling and adipose tissue-specific methylation analysis for study of epigenetic changes related to body mass index (BMI) and suggested that the analysis of blood DNA methylation is worthwhile and can reflect changes in relevant tissues for a phenotype [9].

Circulating insulin-like growth factor binding protein-1 (IGFBP-1) is produced in the liver and is regulated mainly by insulin. This protein binds to insulin-like growth factors (IGF-I and IGF-II), acts as a shuttle of IGFs to target tissues, and regulates the activity of free IGF-I [10]. IGFBP-1 is considered to be the principal acute regulator of IGF-I bioactivity and plays an important role in the development of diabetes and complications [11]. Clinical investigations have demonstrated that low circulating levels of IGFBP-1 are associated with type 2 diabetes (T2D) [12-14], whereas high serum IGFBP-1 levels are associated with T1D [15,16]. Furthermore, serum IGFBP-1 levels are found to be increased in T1D patients with microalbuminuria [17]. According to our previous study in Swedish middle-aged and elderly twins, heredity estimates were only 36% for serum IGFBP-1 levels, which implied that nongenetic factors may contribute to the varied IGFBP-1 levels in diabetes [18]. *IGFBP1* gene is located on chromosome 7p12.3, and a CpG island resides at the promoter and 5′-UTR region in this gene. We recently reported that increased DNA methylation levels are associated with T2D in a Swedish population [19]. In this study, we further analyzed DNA methylation changes of *IGFBP1* in Swedish T1D patients with and without DN and aimed to evaluate the epigenetic changes of this gene in T1D and DN.

## Results

### *IGFBP1* DNA methylation changes and serum protein variation between NGT and T1D

**Figure 1:**
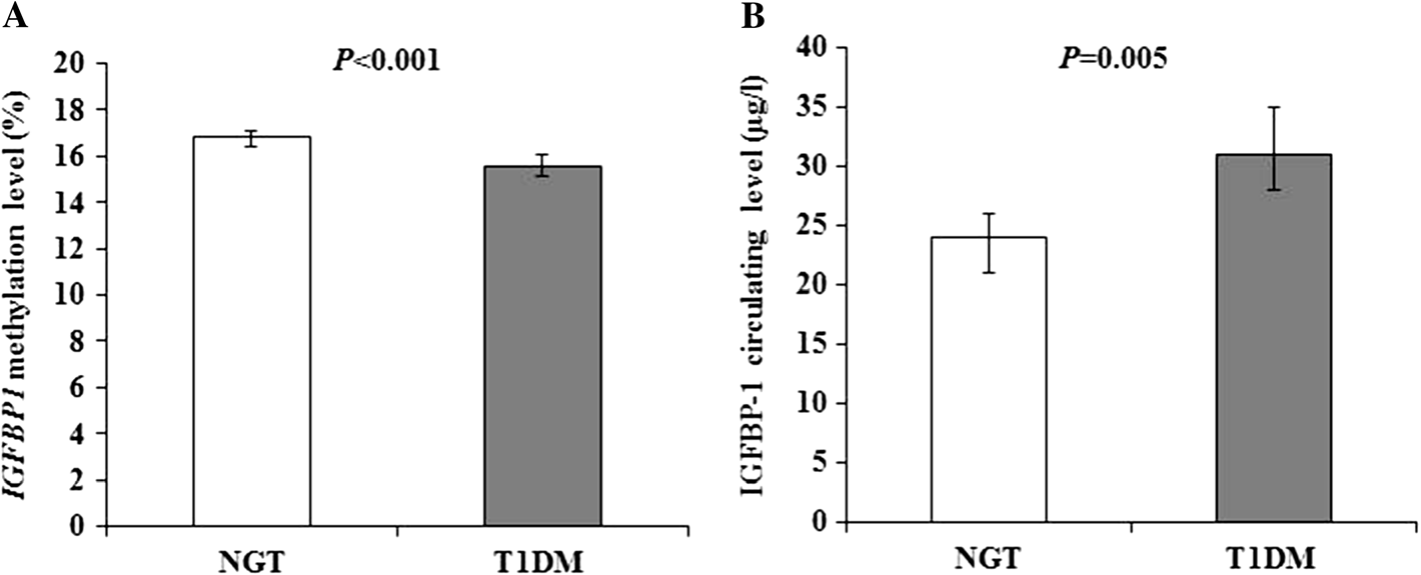
***IGFBP1 *DNA methylation changes and serum protein variation in Swedish men with normal glucose tolerance and type 1 diabetes. (A)** The mean levels of *IGFBP1* DNA methylation in T1D patients were significantly lower than those in NGT subjects (15.6% versus 16.9%; *P* < 0.001, adjusted for age). (**B)** IGFBP-1 serum levels in T1D patients were increased compared with subjects with NGT (31 μg/L versus 24 μg/L; *P* = 0.005).

We analyzed DNA methylation levels at six CpG sites (referred to as P1 to P6) of the *IGFBP1* gene and found that the *IGFBP1* DNA methylation levels at five of the six CpG sites were significantly decreased in T1D patients (P1, 16.8%; P2, 14.4%; P3, 11.1%; P4, 11.9%; P6, 19.4%), compared with those in NGT subjects (P1, 17.7%; P2, 15.6%; P3, 12.7%; P4, 13.8%; P6, 22.2%; *P* = 0.004 for P1, *P* < 0.001 for P2, P3, P4, P6), except at P5 (19.7% in T1D versus 19.5% in NGT; *P* = 0.186). Combining all six CpG sites, the mean levels of *IGFBP1* DNA methylation in T1D patients were significantly lower than those in NGT subjects (15.6% versus 16.9%; *P* < 0.001, Figure 1A). On the contrary, IGFBP-1 serum levels in T1D patients were increased compared with those in subjects with NGT (31 μg/L versus 24 μg/L; *P* = 0.005, Figure 1B).

**Figure 2:**
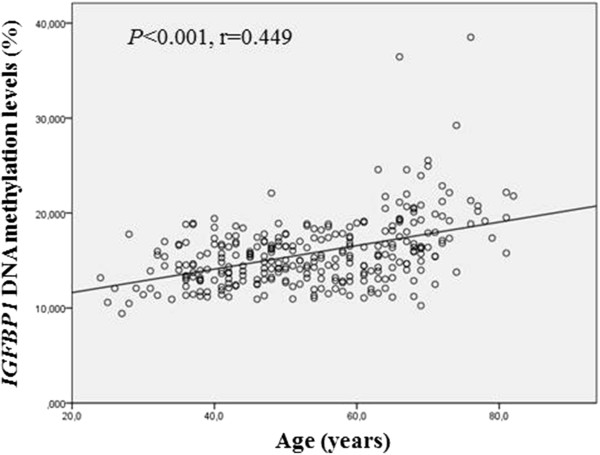
**Correlation of *IGFBP1 *DNA methylation levels with ages in Swedish type 1 diabetes patients.** A positive correlation appeared between *IGFBP1* DNA methylation levels and ages in T1D patients (*r* = 0.449; *P* < 0.001).

We also analyzed the relation of *IGFBP1* DNA methylation levels with ages and circulating IGFBP-1 levels. A positive correlation was found between *IGFBP1* DNA methylation levels and ages in T1D patients (*r* = 0.449; *P* < 0.001) (Figure 2). Similarly, a correlation was seen between *IGFBP1* DNA methylation levels and duration of diabetes in T1D patients (*r* = 0.240; *P* < 0.001) and T1D patients with or without DN (*r* = 0.580 and 0.425; *P* < 0.001 for both). Based on this observation, the comparison analyses of *IGFBP1* DNA methylation levels and *P* values, as described earlier, were adjusted by ages. However, no correlation was found between *IGFBP1* DNA methylation and circulating IGFBP-1 levels in T1D patients with or without DN.

### *IGFBP1* DNA methylation changes and serum protein variation between T1D with and without DN

**Figure 3:**
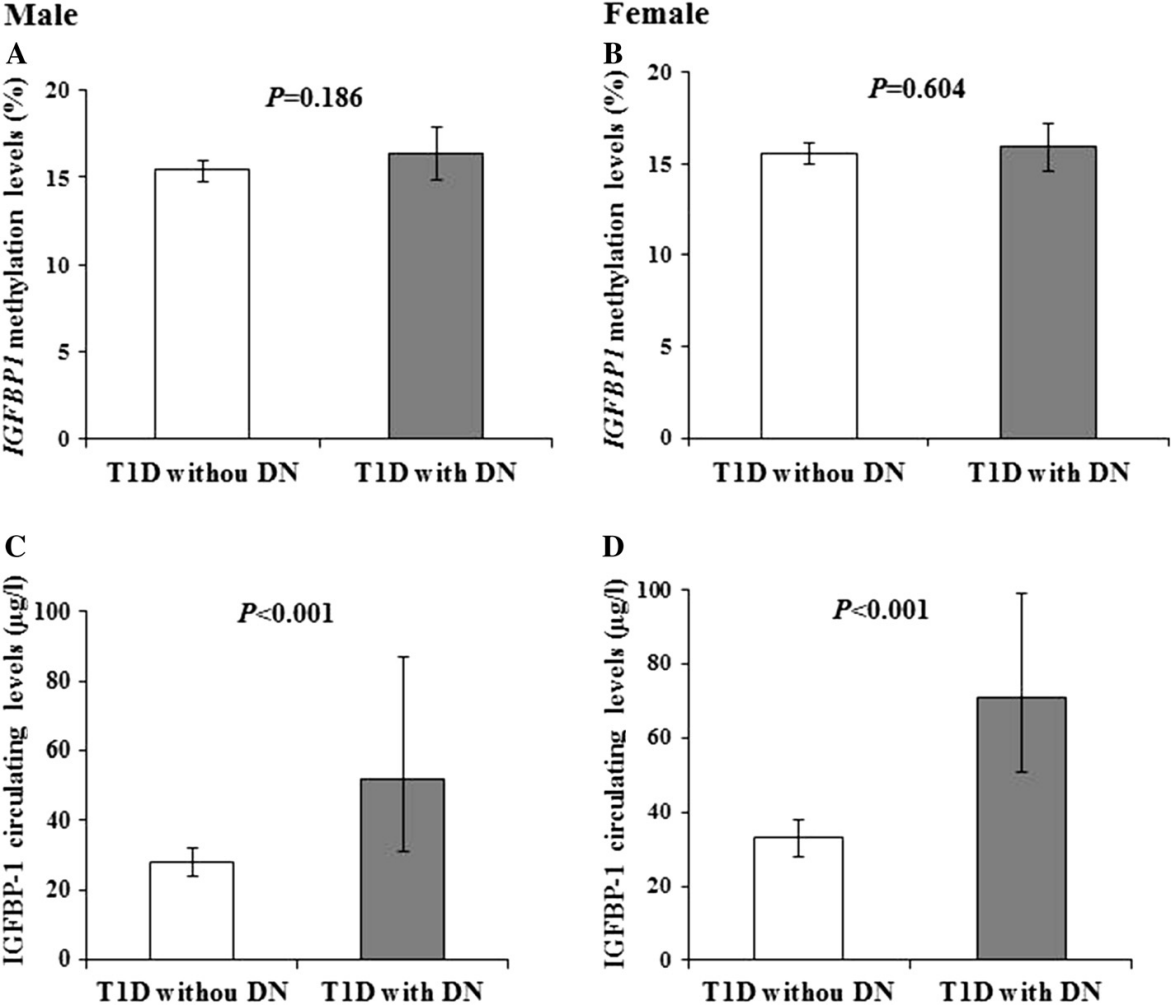
***IGFBP1 *DNA methylation changes and serum protein variation in Swedish type 1 diabetes patients with and without diabetic nephropathy. A** and **B** demonstrated the similar *IGFBP1* DNA methylation levels between T1D with and without DN in either male (16.4% versus 15.4%; *P* = 0.186 adjusted for age) or female patients (15.9% versus 15.6%, *P* = 0.604, adjusted for age). **C** and **D** indicate that the IGFBP-1 serum levels in T1D patients with DN were significantly increased in both male (52 μg/L versus 28 μg/L; *P* = 0.021) and female patients (71 μg/L versus 33 μg/L; *P* = 0.003), in comparison with T1D patients without DN.

We further analyzed *IGFBP1* DNA methylation changes and serum protein levels in T1D patients with and without DN. No difference appeared in *IGFBP1* DNA methylation levels between T1D with and without DN in either males (16.4% versus 15.4%, *P* = 0.186; Figure 3A) or female patients (15.9% versus 15.6%; *P* = 0.604; Figure 3B). Compared with T1D patients without DN, however, the IGFBP-1 serum levels in T1D patients with DN were significantly increased in both male (52 μg/L versus 28 μg/l, *P* = 0.021, Figure 3C) and female patients (71 μg/L versus 33 μg/L; *P* = 0.003; Figure 3D). Furthermore, no difference of serum IGFBP-1 levels was found between male and female patients in T1D with DN (52 μg/L versus 71 μg/L; *P* = 0.17) or the patients without DN (28 μg/L versus 33 μg/L; *P* = 0.28).

### *IGFBP1* DNA methylation changes and serum protein variation according to BMI

We recently demonstrated that *IGFBP1* DNA methylation changes are not dependent on BMI in T2D [19]. To evaluate whether *IGFBP1* DNA methylation changes and serum protein levels were dependent on BMI in T1D, we divided all T1D patients into four groups according to BMI (that is, low-weight group (BMI < 18.5 kg/m^2^), normal-weight group (18.5 to 25.0 kg/m^2^), overweight group (25.0 to 30.0 kg/m^2^), and obese group (≥ 30.0 kg/m^2^). We found that circulating IGFBP-1 levels were decreased gradually according to BMI (51, 39, 30, and 28 μg/L; *P* = 0.012). However, the *IGFBP1* DNA methylation levels were similar among the four groups (16.0%, 15.9%, 15.6%, and 16.0%; *P* = 0.867).

## Discussion

We conducted an epigenetic study of the *IGFBP1* gene in a Swedish cohort and demonstrated that *IGFBP1* DNA methylation levels are decreased in T1D patients compared with NGT subjects. The findings in the present study together with our recent report [19] are summarized in Table 1 and showed that increased and decreased *IGFBP1* DNA methylation levels are associated with T2D and T1D, respectively, implying that *IGFBP1* may confer different epigenetic effects in T1D and T2D. Both T1D and T2D are characterized by hyperglycemia, but the underlying pathogenic mechanisms are different. T1D develops on the basis of autoimmune destruction of pancreatic beta cells, which results in insulin deficiency, whereas the hyperglycemia in T2D results from a combination of impaired insulin secretion and insulin resistance.

**Table 1:** **A summary of the *IGFBP1 *** **DNA methylation and IGFBP-1 serum levels in Swedish men with normal glucose tolerance, type 1 and type 2 diabetes**

**Group**	**Number**	***IGFBP1 *** **DNA methylation levels (%)**	***P *** **value**	**IGFBP-1 serum levels (μg/L)**	***P *** **value**	**Reference**
**T2D**	164	20.0 (19.5-20.5)	*P* < 0.001	18 (16-20)	0.014	Gu T *et al*. 2013 [19]
**NGT**	242	16.9 (16.4-17.1)	*-*	24 (21–26)	-	
**T1D**	304	15.6 (15.1-16.1)	*P* < 0.001	31 (28–35)	0.007	Present study

Data are presented as means (95% CI). NGT, normal glucose tolerance; T1D, type 1 diabetes; T2D, type 2 diabetes; *P* values were from comparison tests of NGT versus T1D or T2D and adjusted for age.

Although our studies have provided the evidence that *IGFBP1* has different DNA methylation levels in T1D and T2D, the molecular mechanism is still unknown. In this study, serum levels of IGFBP-1 in T1D are increased compared with those in NGT subjects, which is consistent with a previous report [15]. The hepatic production of IGFBP-1 protein is regulated mainly by insulin [20]. Therefore, the reduction of IGFBP-1 in T1D is thought to be caused by the insulin deficiency in the patients, although in T2D patients with short duration, the decreased IGFBP-1 concentrations are mainly due to hyperinsulinemia. Thus, we hypothesized that the difference of *IGFBP1* DNA methylation levels between T1D and T2D may be related to insulin activity.

DNA methylation levels are known to be affected by age and gender in healthy and disease conditions. The significant interindividual differences in peripheral blood DNA methylation have been discovered in longitudinal studies, with both increase and decrease of the global genome methylation/specific gene methylation in aging [21,22]. In our recent report, age-matched NGT subjects and T2D patients were included, and the range of their ages was limited [19]. In the present study, we found a positive relation between *IGFBP1* DNA methylation levels and ages in T1D patients. Although similar correlation between *IGFBP1* DNA methylation levels and T1D duration was observed, the onset of T1D occurred mainly in childhood, so we believe this correlation was affected mainly by age. The mechanism behind the *IGFBP1* DNA methylation with aging is still unknown. Aging-related insulin resistance in T1D could be one of the explanations. In addition, Benbassat *et al.*[23] showed that serum IGFBP-1 levels were slightly increased throughout adulthood. However, we could not find any correlation between serum IGFBP-1 levels and ages in T1D patients.

In the present study, we showed that both male and female patients with DN had increased IGFBP-1 serum levels compared with those without DN. Flyvbjerg *et al*. [24] previously conducted experiments with diabetic rats and demonstrated that kidney accumulation of IGF-1 is associated with kidney hypertrophy, which is an early feature of DN. The increased circulating IGFBP-1 allows trapping IGF-1 accumulated in kidneys and may consequently contribute to the development of DN.

Consistent with our recent report of *IGFBP1* DNA methylation changes in T2D [19], data from the present study imply that *IGFBP1* DNA methylation levels are not dependent on BMI. Furthermore, no correlation between *IGFBP1* DNA methylation changes and serum IGFBP-1 levels were observed. Several reasons may be included in the explanation. First, multiple regulators exist for IGFBP-1 circulating levels, including stimulators such as glucagon, cytokines, and cortisol, or suppressors like insulin and amino acids [10,20,25,26]. Second, DNA samples used for methylation analyses were extracted from peripheral blood cells. A limitation in the present study is that we are unable to perform liver tissue-specific methylation analysis in T1D patients and NGT subjects. Third, DNA methylation changes may affect mRNA transcription directly, but not the protein-synthesis process. Although a recent study indicated that DNA methylation analysis with whole-blood samples can be used to reflect the changes in relevant tissues [9], it may be interesting to investigate DNA methylation and mRNA expression levels of the *IGFBP1* gene in liver, in order to better understand the correlation between DNA methylation changes and serum protein levels.

## Conclusion

In conclusion, this study demonstrates for the first time that the decreased *IGFBP1* DNA methylation levels are associated with T1D but not with DN and contributes further information that increased circulating IGFBP-1 levels are associated with T1D and related DN.

## Methods

### Subjects

This is a case–control study. In total, 778 Swedish subjects were enrolled, consisting of 536 T1D patients (304 male/232 female) as diabetic cases and 242 men with normal glucose tolerance (NGT). According to the World Health Organization criteria [27], all patients with T1D were diagnosed before 31 years of age. The duration of T1D was more than 10 years. The NGT subjects were selected from Stockholm Diabetes Prevention Programs (SDPP), as described previously [19].

Clinical characteristics of all subjects with NGT or T1D were summarized in the Additional file 1: Table S1. Urinary AER, 20 to 200 μg/min in at least two consecutive overnight samples was considered microalbuminuria, whereas AER > 200 μg/min in at least two consecutive overnight samples was considered macroalbuminuria. The 51 (25 male/26 female) T1D subjects with macroalbuminuria were classified as the cases of T1D with DN, including two T1D patients who received renal-replacement therapy, whereas 296 (160 male/136 female) T1D patients with persistent normal albuminuria were grouped as the controls of T1D without DN. In addition, 189 (119 male/70 female) T1D patients with normal albuminuria or historic microalbuminuria had medical treatments with angiotensin-converting-enzyme inhibitor (ACEI)/angiotensin II-receptor blockers (ARBs). We excluded these 189 patients when we performed comparison analyses between T1D patients with DN and those without DN. Clinical characteristics of all T1D patients with or without DN are represented in Additional file 2: Table S2.

Informed consent was obtained from all subjects, and the study was approved by the ethics committee of the Karolinska Institutet, Stockholm, Sweden.

### Serum IGFBP-1 and IGF-1 measurements

Serum samples were collected from all T1D subjects between 10 AM and 3 PM, and fasting serum samples were collected from NGT subjects. All serum samples were saved at −80°C until measurements. We used an in-house RIA, as described previously, to measure serum IGFBP-1 levels [12]. The sensitivity was 3 *μ*g/L, and the intra- and interassay CVs were 3% and 10%, respectively. We also measured total serum IGF-1 levels with another in-house RIA after the separation of IGFs from IGFBPs by acid ethanol extraction and cryoprecipitation [28]. To minimize the interference of remaining IGFBPs, des [1-3] IGF-1 was used as a tracer. The intra- and interassay CVs were 4% and 11%, respectively.

### DNA methylation analysis of *IGFBP1*

Genomic DNA was extracted from peripheral blood samples in all subjects. We used the same bisulfite pyrosequencing protocol as recently described in our epigenetic study of the *IGFBP1* gene in T2D [19]. This protocol has been widely used for sensitive and quantitative universal pyrosequencing methylation analysis of CpG sites [29]. DNA methylation levels at six CpG sites at 5′-UTR of the *IGFBP1* gene were determined by using PyroMark CpG assay (ENSG00000146678), a PyroMark PCR kit (Qiagen, Hilden, Germany) and PyroMark Q96 ID pyrosequencing system (Biotage, Uppsala, Sweden). To control the conversion efficiency of the bisulfite treatment and accuracy in methylation analyses, unmethylated bisulfite converted and unconverted DNA samples (Qiagen) were used.

### Statistical analyses

All data were analyzed by using PASW statistics program (SPSS 20.0; Chicago, IL, USA). Data presented in the tables and figures are either mean with 95% confidence interval (CI) or geometric mean with 95% CI if the data were not normally distributed. Continuous variables between groups were compared by using an unpaired *t* test or one-way ANOVA followed with Tukey *post hoc* test. Covariate-adjusted generalized linear models were used when adjusting for age. Linear regression analyses were used to examine the relation between variables. *P* values < 0.05 were considered statistically significant.

## Competing interest

The authors declare that no competing interest is associated with this article.

## Authors’ contributions

TG, HFG, and KB designed the study. HF and KB collected the subjects and clinical data. TG performed experiments and analyzed the data. TG, HFG, and KB wrote the first draft of the manuscript. All authors contributed to the interpretation of the data and critical revision of the manuscript. All authors read and approved the final manuscript.

## Supplementary Material

Additional file 1: Table S1 Clinical characteristics of Swedish subjects with normal glucose tolerance and type 1 diabetes. Click here for file

Additional file 2: Table S2 Clinical characteristics of Swedish type 1 diabetes patients with and without diabetic nephropathy. Click here for file
