# Supplementary material for: Epigenetic analyses of the insulin-like growth factor binding protein 1 gene in type 1 diabetes and diabetic nephropathy
Source: Clin Epigenetics. 2014 May 30;6(1):10. doi: 10.1186/1868-7083-6-10 (PMC4046502; doi:10.1186/1868-7083-6-10)
Supplement: Additional file 1: Table S1 — Clinical characteristics of Swedish subjects with normal glucose tolerance and type 1 diabetes. [file 1868-7083-6-10-S1.pdf]

Supplemental table 1. Clinical characteristics of Swedish subjects with normal glucose tolerance and type 1 diabetes

	NGT	T1D
N (Male/Female)	242 (242/0)	536 (304/232)
Age (years)	58 (57-58)	50 (48-51)/50 (48-52)
BMI (kg/m^2)	26.1(25.9-26.3)	25.9 (25.5-26.3)/25.2 (24.6-25.7)
SBP (mmHg)	135 (134-138)	131 (130-132)/128 (126-130)
DBP (mmHg)	83 (82-84)	75 (74-76)/72 (70-73)
f-Glucose (mmol/L)	5.0 (4.9-5.1)	-

Data were expressed as means (95%CI). NGT: normal glucose tolerance; T1D: type 1 diabetes; BMI: body mass index; SBP and DBP: systolic and diastolic blood pressures.
