# Supplementary material for: Epigenetic analyses of the insulin-like growth factor binding protein 1 gene in type 1 diabetes and diabetic nephropathy
Source: Clin Epigenetics. 2014 May 30;6(1):10. doi: 10.1186/1868-7083-6-10 (PMC4046502; doi:10.1186/1868-7083-6-10)
Supplement: Additional file 2: Table S2 — Clinical characteristics of Swedish type 1 diabetes patients with and without diabetic nephropathy. [file 1868-7083-6-10-S2.pdf]

Supplemental table 2. Clinical characteristics of Swedish type 1 diabetes patients with and without diabetic nephropathy

	**T1D-DN**	**T1D+DN**	***P* value**
N (Male/Female)	296 (160/136)	51 (25/26)	
Age (years)	44 (43-46)	57 (53-61)	<0.001
Years since diagnosis	25 (24-27)	41 (38-45)	<0.001
BMI (kg/m^2)	25.2 (24.8-25.6)	26.3 (24.9-27.7)	0.063
HbA_{1C} (%)	8.1 (8.0-8.2)	8.4 (8.0-8.8)	0.087
SBP (mmHg)	125 (123-127)	136 (131-141)	<0.001
DBP (mmHg)	74 (73-75)	71 (68-74)	0,023
Triglycerides (mg/dl) *	62.0 (58.5-65.4)	110.0 (91.8-131.9)	<0.001
Total cholesterol (mg/dl)	188.0 (184.0-192.0)	175.3 (162.6-188.1)	0.023
LDL (mg/dl)	110.1 (106.7-113.5)	94.0 (82.3-105.8)	<0.001
HDL (mg/dl)	64.1 (61.9-66.4)	56.1 (49.3-62.9)	0.010
ACR (mg/g)	6.7 (5.6-7.8)	332.1 (233.8-431.4)	<0.001
Creatinine (mg/dl)	0.81 (0.79-0.83)	1.88 (1.50-2.26)	<0.001
eGFR (mL/min/1.73m^2)	100.7 (98.9-102.5)	49.0 (40.7-57.3)	<0.001
IGF-1 (µg/L)*	112 (106-119)	115 (100-131)	0.798
IGF-1 SD score	-1.9 (-2.1-1.7)	-1.0 (-1.5-0.5)	0.003

T1D: type 1 diabetes; DN: diabetic nephropathy; BMI: body mass index; SBP and DBP: systolic and diastolic blood pressures; LDL and HDL: low and high density lipoproteins; ACR: albumin/creatinine ratio; eGFR: estimated glomerular filtration rate (calculated by CKD-EPI creatinine equation).

Data were expressed as means (95%CI) for normally distributed variables and as geometric means (95% CI) for *non-normally distributed variables; Serum IGF-I standard deviation (SD) score = [(10 log IGF-$I_{observed}$+0.00693*age)-2.581]/0.120.
